# The history of entamoebiasis

**DOI:** 10.1017/S0031182025100279

**Published:** 2025-05

**Authors:** Dietmar Steverding

**Affiliations:** Norwich Medical School, University of East Anglia, Norwich, UK

**Keywords:** amoebiasis, amoebic dysentery, amoebic liver abscess, *Entamoeba dispar*, *Entamoeba histolytica*, entamoebiasis

## Abstract

This review article summarizes the history of amoebic dysentery (entamoebiasis) caused by *Entamoeba histolytica*. Initially, *Entamoeba* species were thought to be the most primitive extant eukaryotes, but more recent research revealed that they emerged relatively late in evolutionary history. Paleoparasitological data suggest that *E. histolytica* has been a parasite of humans since ancient times and was probably spread throughout the world by man during early human migration. By the end of the 19th century, it was established that *E. histolytica* was the etiological agent of amoebic dysentery and liver abscess. The issue over pathogenic and non-pathogenic strains of *E. histolytica* was resolved in the 1980s by the discovery of the morphologically indistinguishable harmless sister species *Entamoeba dispar*. Being mainly a disease of tropical and subtropical low-income countries, entamoebiasis cases have increased among travellers and immigrants arriving from endemic regions in recent years.

## Graphical abstract

**Figure:**
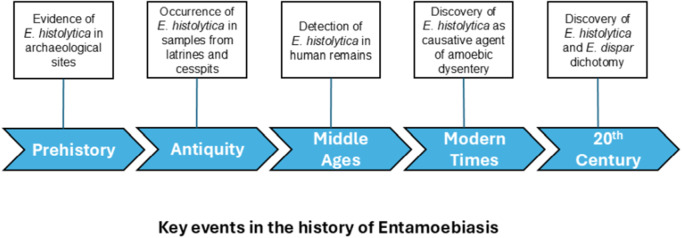

## Introduction

Diarrhoeal diseases have been and still are one of the leading causes of death (Hénock Blaise and Dovie, 2007). The causes of diarrhoeal diseases are manifold and include viral, bacterial and parasitic pathogens. The protozoan *Entamoeba histolytica* is the etiological agent of amoebic dysentery (entamoebiasis) in humans. The amoeba is found worldwide; however, the majority of entamoebiasis cases occur in developing countries. Each year, about 50 million people contract the infection, and approximately 100 000 individuals die from the disease (Zulfiqar et al., 2023).

The disease is transmitted orally via ingesting mature *E. histolytica* cysts in faecally contaminated water or food, or from hands. In the small intestine, the cysts encyst and release trophozoites, which move into the large intestine. There, the trophozoites replicate by binary fission and form new cysts. Both trophozoites and cysts are passed with the faeces, although trophozoites are typically excreted with loose stools while cysts are released with formed excrements. Due to the protective wall, cysts can survive and remain infectious for up to 12 days in moist and cool environments and up to 30 days in water (Sanie et al., 2012), whereas trophozoites die rapidly outside the body. In most cases, trophozoites occur in the intestinal lumen (non-invasive infection or luminal entamoebiasis), causing no apparent disease manifestation (asymptomatic infection). In about 10–20% of cases (Uribe-Querol and Rosales, 2020), the trophozoites invade the mucosal lining of the intestine (intestinal disease), inducing amoebic colitis with severe abdominal pain, diarrhoea (which may contain blood and mucus) and fever. In rare cases, *E. histolytica* can cross the intestinal barrier and spread via the blood to infect other organs (extraintestinal entamoebiasis), leading to abscesses. The most common extraintestinal manifestation is the amoebic liver abscess. In rare cases, an amoebic liver abscess can rupture into the abdomen or chest, causing the formation of abscesses in the lungs and heart. Extraintestinal manifestation is a severe complication reaching mortality rates exceeding 20% in cases of amoebic pericarditis and pulmonary entamoebiasis. However, if intestinal entamoebiasis is treated appropriately, the prognosis is good and the mortality rate is less than 1% (Zulfiqar et al., 2023).

## Evolution of *E. histolytica*

The genus *Entamoeba* was previously considered to be the most primitive extant eukaryotic group (Clark, 2000; Cui et al., 2019). Because of atypical subcellular structures (lack of mitochondria, rough endoplasmic reticulum and Golgi apparatus) and an unusual glycolytic metabolism (pyrophosphate-dependent glycolytic pathway enzymes) and based on phylogenetic analysis of the elongation factor-1α, it was suggested that entamoebae had diverged from the line of eukaryotes with mitochondria prior the endosymbiotic event of a protomitochondrion (Hasegawa et al., 1993; Clark, 2000). For these reasons, entamoebae were regarded as the earliest eukaryotic cells and thus living relics. However, more recent research revealed that entamoebae do not represent the ancestral form of eukaryotic cells. There is more and more evidence that entamoebae emerged relatively late in evolution and that the absence of typical eukaryotic organelles has arisen through secondary loss during lifestyle adaptation. Direct evidence for the secondary loss of mitochondria in entamoebae came from identifying genes encoding proteins of mitochondrial origin (Clark and Roger, 1995; Arisue et al., 2002; van der Giezen et al., 2005). The discovery of a previously undescribed organelle of mitochondrial origin termed mitosome, to which the nuclear-encoded mitochondrial genes are targeted, suggests that entamoebae retained a greatly reduced version of the mitochondrion (Mai et al., 1999; Tovar et al., 1999). The demonstration of a continuous endoplasmic reticulum is further evidence that entamoebae developed quite late in evolution (Teixeira and Huston, 2008). In addition, small-subunit ribosomal RNA (ssrRNA) phylogenetic analysis revealed that entamoebae emerged more recently than several lineages of typical eukaryotes with mitochondria (Sogin, 1991; Cavalier-Smith, 1993).

**Figure 1.**
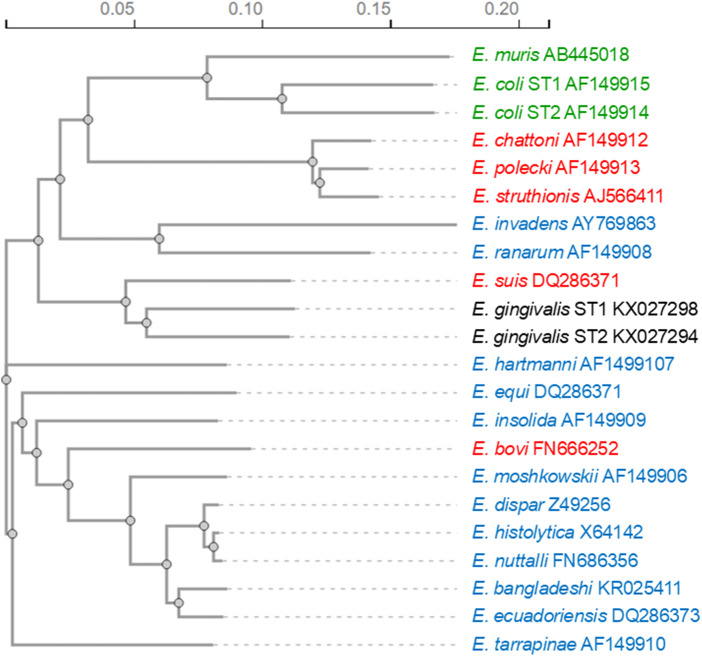
Phylogenetic relationship among *Entamoeba* species based on small-subunit ribosomal RNA sequences. The phylogenetic tree was calculated using the neighbour-joining method with the programme Clustal Omega (Madeira et al., 2024). Species producing cysts with 8 nuclei are highlighted in green, those producing cysts with 4 nuclei in blue and those producing cysts with 1 nucleus in red. Species producing no cysts are shown in black.

The genus *Entamoeba* is classically divided into three groups of species that form cysts with 1, 4 or 8 nuclei and one group of species that do not produce cysts. Previous ssrRNA sequence analyses seemed to verify the validity of the grouping of the cyst-forming species (Clark and Diamond, 1997; Silberman et al., 1999). The clustering of non-encysting species with the group of species forming cysts with 4 nuclei was taken as an indication that this trait was acquired due to secondary loss (Clark and Diamond, 1997). However, more recent phylogenetic relationship analyses revealed that newly discovered mononucleate cyst-producing species (*E. bovis* and *E. suis*) group with the clades of quadrinucleate cyst-producing species (Figure 1) (Clark et al., 2006; Stensvold et al., 2010). This indicates that these mononucleated cyst-producing species are descended from quadrinucleate cyst-producing species (Stensvold et al., 2010). Phylogenetic studies also suggest that *Entamoeba* sp. producing cysts with 8 nuclei form the basal group, whereas those species making 4-nucleated cysts constitute the most derived group (Silberman et al., 1999), which includes the human pathogen *E. histolytica*.

*Entamoeba histolytica* groups with the clade including *E. nuttalli, E. dispar, E. bangladeshi* and *E. ecuadoriensis* (Figure 1). The closest relative of *E. histolytica* is *E. nuttalli*, a non-pathogenic amoeba prevalent in wild and captive macaques (Tachibana et al., 2007; Cui et al., 2019). The second phylogenetically closest species to *E. histolytica* is *E. dispar*, which is morphologically not differentiable from *E. histolytica* but non-pathogenic to humans. The morphological indistinguishability of the two species was the reason why previous prevalence data of entamoebiasis were greatly overestimated, with *E. dispa*r infections being 10 times more common than *E. histolytica* infections (Gonin and Trudel, 2003). Of the other two species of the *E. histolytica* clade, *E. bangladeshi* is also morphologically indistinguishable from *E. histolytica* but is considered non-pathogenic (Royer et al., 2012; Cui et al., 2019), while *E. ecuadoriensis* was isolated from sewage; therefore, it remains unclear whether it is a free-living or parasitic species (Clark et al., 2006).

The analysis of the genome of *E. histolytica* revealed some interesting insights into the evolution of this pathogen. Noteworthy is the elimination of metabolic enzymes by secondary loss, including the loss of the Krebs cycle and respiratory chain proteins (Loftus et al., 2005). Genomic data also confirm the absence of a mitochondrial genome, which supports the finding that *E. histolytica* lacks a classical mitochondrion and possesses only a mitochondrion-derived mitosome (Loftus et al., 2005). On the other hand, the amoeba has gained a considerable number of metabolic enzyme-encoding genes (at least 68) via horizontal gene transfer from bacteria (Weedall and Hall, 2011; Das and Ganguly, 2014). Among these bacterial genes are those coding for fermenting enzymes driving the anaerobic metabolism of *E. histolytica* (Clark et al., 2007). The acquisition of most of these bacterial genes seems to be ancient as several orthologous genes have also been found in the distant relative *E. invadens* (Roy et al., 2006). The horizontal transfer of bacterial genes and the instability and plasticity of the genome of entamoebae have been important for the adaptive evolution of *E. histolytica* (Weedall and Hall, 2011).

## Historical evidence of *E. histolytica*

There is global evidence for the presence of *E. histolytica* in historical times. By using ELISA techniques to determine *E. histolytica*-specific antigens, the amoeba was detected in specimens from coprolites, latrines, cesspits and human remains.

### Prehistoric times

The earliest evidence of modern strains of *E. histolytica* comes from sediment samples of archaeological sites in Switzerland, Greece and France, dating back to 5700–2500 BP (Goncalves et al., 2004; Le Bailly and Bouchet, 2006, 2015). Thus, identification of *E. histolytica* in prehistoric Western and Southern Europe may suggest that entamoebiasis could have originated in the Old World in the Neolithic period (Le Bailly et al., 2016). However, since modern strains of *E. histolytica* were also detected in specimens from human remains and cesspits in the Americas dating back to pre-Columbian times (Fouant et al., 1982; Goncalves et al., 2004; Le Bailly et al., 2014), entamoebiasis has probably developed much earlier in human evolution.

### Antiquity

There are several detailed accounts of diarrhoeal diseases by ancient Chinese, Hebrew, Greek and other chroniclers, but given the unspecific symptoms of dysentery, these reports do not necessarily describe entamoebiasis (Imperato, 1981). Nevertheless, the occurrence of *E. histolytic* infections in antiquity was confirmed by ELISA in samples from latrines, cesspits, pits and sediments collected from 6 archaeological sites in Europe (Belgium, France and Italy) dating from the Roman period (Goncalves et al., 2004; Le Bailly and Bouchet, 2006, 2015). In another sample from an occupation layer from an archaeological site in Israel of the same period, amoebic cysts attributed to *E. histolytica* were described microscopically (Witenberg, 1961). As the finding was solely based on microscopy, it is rather speculative whether the observed structures were indeed *E. histolytica* cysts (Le Bailly et al., 2016).

### Middle ages

The presence of *E. histolytica* during Medieval times in Europe was confirmed by ELISA analysis of samples from latrines/cesspits, human remains, occupation layers and a tomb of 6 archaeological sites in Belgium, France, Latvia and Switzerland (Goncalves et al., 2004; Le Bailly and Bouchet, 2006, 2015; Yeh et al., 2014; Graff et al., 2020). The protozoan parasite was also detected in 2 locations in Israel in latrine/cesspit samples dating back to the Crusader and Mamluk periods (Mitchell et al., 2008; Yeh et al., 2015). These findings may indicate that *E. histolytica* was introduced into medieval Israel by travellers from Europe (Le Bailly et al., 2016).

In the New World, the presence of *E. histolytica* in pre-Columbian times was potentially detected at four locations. In Chile and Peru, entamoebae were microscopically observed on mummies (Fouant et al., 1982). Since these findings were not confirmed by immunological techniques, it cannot be assumed beyond doubt that the observed cysts are from *E. histolytica*. However, a more recent study on a pre-Columbian human skeleton from Guadeloupe in the Caribbean recovered the protozoan parasite and, thus, proved that *E. histolytica* was present in the Americas before European colonization (Le Bailly et al., 2014). Also, samples from cesspits at an archaeological site in North America dating back to the 12th–13th century tested positive for the presence of *E. histolytica* antigens (Goncalves et al., 2004). These latter two results indicate that *E. histolytica* was most likely introduced into the New World during the prehistoric population of the continent, similar to the spreading of soil-transmitted helminths to the Americas (Steverding, 2020).

### Modern era

*Entamoeba histolytica* was detected in latrine/cesspit samples of two archaeological sites in Belgium and France, dating back to the 17th–18th century (Goncalves et al., 2004; Le Bailly and Bouchet, 2015). At the same period, the parasite was also identified on a human skeleton from a cemetery in Guadeloupe (Le Bailly et al., 2014). As for the 19th century, the amoeba was discovered in samples from the Meadowlark cemetery in the USA, from cesspits in Argentina and on a human skeleton in Guadeloupe (Goncalves et al., 2004; Le Bailly and Bouchet, 2006; Le Bailly et al., 2014).

The 19th century was the era when *E. histolytica* was discovered and identified as the causative agent of amoebic dysentery and amoebic liver abscess. In the earlier part of the century, several British physicians gave accounts of dysentery associated with liver disease in India. In 1818, the Scottish physician and surgeon Sir George Ballingall (1780–1855) published a book on fever, dysentery and liver complaints among European troops in India, in which he described ‘*… the existence of a liver affection in most of the cases of colonitis …*’ (Ballingall, 1818). Similar observations were made by the Irish surgeon Sir James Annesley (1774–1847) during his time at the Madras General Hospital, which he published in a 2-volume book in 1828. He described the disease as ‘*hepatic dysentery*’ but was unsure about the root cause of the disease: the liver abscess or the dysentery (Annesley, 1828). Nevertheless, from the clinical symptoms Annesley recorded, it is clear that he was describing entamoebiasis. Four years later, the Scottish surgeon William Twining (1790–1835), while working as commander-in-chief of the Indian army in Bengal, confirmed Annesley’s observations (Twining, 1832). In 1846, the English physician Edmund Alexander Parkes (1819–1876) published a book connecting liver abscesses with dysentery (Parkes, 1846).

**Figure 2.**
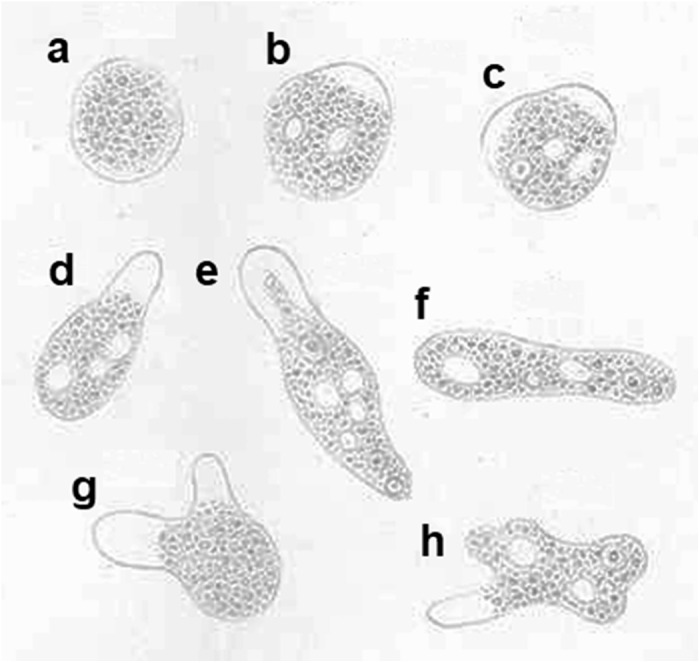
Drawings of amoebas found in the stool of an amoebic dysentery patient investigated by Lösch (Lösch, 1875). (a) Stationary amoeba; (b–h) moving amoebas. (a and g) Neither nucleus nor vacuoles are visible; (c, e, f and h) nucleus and vacuoles are visible; (b and d) only vacuoles are visible; (b, d, e and h) with 1 pseudopod; (c and g) with 2 pseudopods. Magnification, 500X. No permission is needed for reusing Lösch’s drawings as their copyright has expired.

In 1875, the Russian medical doctor Fedor Aleksandrevitch Lösch (alternatively spelt as Fyodor Alexandrovich Lesh) (1840–1903) published probably the first detailed description of a case of amoebic dysentery (Lösch, 1875). The reported case concerned a 24-year-old farmer who was admitted to the clinic of Prof E. Eichwald in St. Petersburg on the 3rd of November 1873. Having had severe diarrhoea for months, the patient was in poor condition. Lösch’s meticulous documentation and thorough analysis of the case were crucial in clarifying the cause of amoebic dysentery. For instance, the regular examination of stools revealed that at times the patient was releasing amoebas on a massive scale. Lösch described the patient’s stools as thin, strong-smelling and red-brown coloured, containing significant amounts of yellowish-white and greyish-coloured lumps of mucus and pus. After 4 months, the patient developed pleuritis accompanied by the disappearance of the amoebas in the stools. Lösch noticed that the vanishing of the amoebas had a positive effect on the enteritis and the consistency of the stools. However, on the 12th of April 1874, the patient eventually died suffering from symptoms of severe anaemia and general exhaustion. The post-mortem inspection revealed that the mucous membrane of the large intestine was thickened and swollen due to the inflammatory infiltration of the submucosal tissue. In his publication, Lösch also described the observed amoebas in great detail, including form and size with intracellular structures, and type of locomotion, and provided detailed drawings of the parasite (Figure 2), which he named *Amoeba coli*. However, Lösch thought that the amoebas were not responsible for the patient’s dysentery but instead inhabited the patient’s intestine later during the disease, triggering the inflammation.

In the following years, several reports were published on the discovery of amoebas in patients with diarrhoeal diseases. During an expedition to investigate the cholera epidemics in Egypt and India of 1883, the German physician and microbiologist Robert Koch (1843–1910) described 5 cases of dysentery, 2 of which included liver abscesses (Koch and Gaffky, 1887). Koch noted numerous amoebas in colonic ulcers, and in tissues and capillaries near hepatic abscesses. Contrary to Lösch, Koch concluded that the amoebas might be responsible for the pathogenesis of the disease. Over the course of 2 years at the General Hospital in Alexandria, Koch’s former student, Stephan Kartulis (1852–1920), observed over 150 cases of diarrhoea patients with amoebas in their stool (Kartulis, 1886). In 1887, the Czech pathologist Jaroslav Hlava (1855–1924) reported that he had detected amoebas in 60 cases of dysentery while working in Prague (Hlava, 1887). The first description of amoebas in a case of dysentery and liver abscess in the New World was by the Canadian physician William Osler (1849–1919) at the Johns Hopkins Hospital in 1890 (Osler, 1890). Several more cases of US patients suffering from dysentery with amoebas in the stools were subsequently published (Imperato, 1981).

In 1891, the American pathologist William Thomas Councilman (1854–1933) and the Canadian physician and parasitologist Henri Amadée Lafleur (1863–1939) published a monograph reviewing 15 cases of amoebic dysentery (Councilman and Lafleur, 1891). The two were the first to recognize that amoebic dysentery is a distinct clinical disease caused by a specific pathogen. They named the causative agent *Amoeba dysenteriae* and coined the terms ‘amoebic dysentery’ and ‘amoebic abscess of the liver’. Performing numerous animal experiments, the German bacteriologist Walter Kruse (1864–1943) and the Italian medical officer Alessandro Pasquale demonstrated in 1894 that amoebic dysentery was directly caused by the amoebas (Kruse and Pasquale, 1894). Priorly, it was thought that the amoebas were opportunistic protozoans that exacerbated existing lesions (Imperato, 1981). Already 1 year earlier, the German internist and surgeon Heinrich Irenaeus Quincke (1842–1922) and the German physician and microbiologist Ernst Roos (1866–1926) described the cyst form of the amoebas and showed that cysts caused dysentery when given orally (Quincke and Roos, 1893).

**Figure 3.**
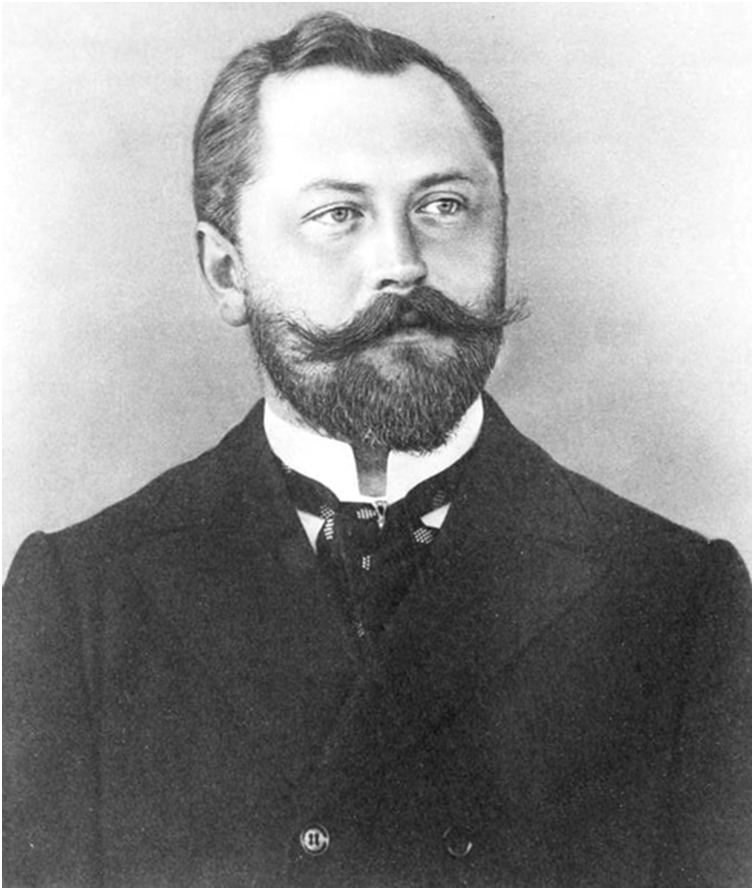
Portrait of Fritz Schaudinn, who proposed the name *Entamoeba histolytica* for the etiological agent of amoebic dysentery. Wikimedia Commons, link: https://upload.wikimedia.org/wikipedia/commons/4/44/fritz_richard_schaudinn.png. Tragically, Schaudinn died in 1906, aged 34, while travelling back to Germany from an International Medicine Meeting in Lisbon, Portugal. He fell suddenly ill with gastrointestinal amoebic abscesses and had to undergo emergency surgery onboard the ship, and eventually died of septicaemia. The amoebic infection was probably self-inflicted (Imperato, 1981).

With the discovery of amoebas as causative agents of dysentery, there came confusion regarding the naming of the pathogen. Whereas the Americans called the dysentery-causing organism *A. dysenteriae*, the Europeans called it *Amoeba coli* (Lösch) or *A. coli felis* (Imperato, 1981). Quincke and Roos proposed the name *Amoeba intestini vulgaris* for a non-pathogenic amoeba found in humans (Quincke and Roos, 1893). In 1897, the Italian microbiologists Oddo Casagrandi (1872–1943) and Pietro Barbagallo (1868-?) also described a non-pathogenic amoeba species and named it *Entamoeba hominis* (Casagrandi and Barbagallo, 1897). Already in previous years, several researchers observed apparently harmless amoebas in patients without dysentery (Imperato, 1981). Both *A. intestini vulgaris* and *E. hominis* are probably identical to *Entamoeba coli*. Eventually, in 1903, it was the German zoologist Fritz Schaudinn (1871–1906; Figure 3) who suggested the name *E. histolytica* for the pathogenic amoeba species because of its ability to destroy tissue and *E. coli* (Lösch) for the harmless amoeba species (Schaudinn, 1903).

Already in 1900, the American tropical medicine specialist Richard P. Strong (1872–1948) differentiated between pathogenic and harmless amoeba species. However, his work remained generally unnoticed (Imperato, 1981). The conclusive demonstration that *E. histolytica* is pathogenic and *E. coli* is non-pathogenic was eventually provided by the American physician Ernest Linwood Walker (1870–1952) and Andrew Watson Sellards (1884–1942) in 1913 (Walker and Sellards, 1913). By administering cysts of the two amoeba species to volunteers at a Manila prison, they were able to show that *E. histolytica* is the aetiological organism of amoebic dysentery in humans and that the encysted stage is the infectious agent. In addition, Walker and Sellards conceived the concept of ‘carrier’, infected individuals constantly passing *E. histolytica* in their stools, thus responsible for spreading the disease (Walker and Sellards, 1913). They also found that infections with *E. histolytica* do not always give rise to clinical disease, suggesting that this amoebic species could act as a commensal, a view rejected by others at that time (Elson-Dew, 1971).

By 1913, the life cycle of *E. histolytica* was finally unravelled by the Dutch parasitologists Willem Abraham Kuenen (1873–1951) and Nicolaas Hendrik Swellengrebel (1885–1970) (Kuenen and Swellengrebel, 1913). They reported that this amoeba species has three life-cycle stages: a quadrinucleate cyst, a commensal *minuta* form and an invasive *magna* form. In 1919, the British protozoologist Clifford Dobell (1886–1949) published a monograph consolidating the current state of knowledge about the amoebas living in man and clarifying their systematics to put order into the conflicting taxonomy of the various amoebic species described thus far (Dobell, 1919). Hence, by the end of the 1910s, the main facts regarding the causative agents of amoebic dysentery, the transmission of the disease and the life-cycle stages of the pathogen, including their food habits and where they live in the human body, were established.

## *E. histolytica/E. dispar* dichotomy

Early on, the low incidence of clinical disease in *E. histolytica* infections raised doubts about whether the amoebic species might generally be a harmless commensal of the human gut flora. It was the French parasitologist Émile Brumpt (1877–1951) who, in 1925, first suggested the existence of 2 morphologically identical but pathogenetically different species (Brumpt, 1925). Brumpt’s conclusion was based on experimental infections of human volunteers and kittens, a very sensitive animal model for determining the pathogenicity of *E. entamoeba*. He named the non-pathogenic species *Entamoeba dispar*. In 1931, the Yugoslavian physician and parasitologist Tshedomir (Čedomir) Simić (1896–1969) provided further evidence for the existence of two morphologically indistinguishable pathogenic and harmless species (Simić, 1931a,b,c). By serially passaging non-invasive amoebas between human volunteers and kittens and vice versa, Simić could show that neither humans nor kittens developed clinical symptoms of amoebic dysentery. However, as Brumpt and Simić could not distinguish the two species morphologically, their description of a non-pathogenic, from *E. histolytica* morphologically indistinguishable amoebic species did not get much support. On the contrary, in 1950, the British protozoologist and parasitologist Cecil Hoare (1892–1984) published a review article that cemented the view that the majority of cases of entamoebiasis are infections with non-pathogenic *E. histolytica* strains, while occasionally virulent strains of the amoeba lead to amoebic dysentery (Hoare, 1950).

The idea of two species gained momentum again in 1978 when it was reported that pathogenic and non-pathogenic *E. histolytica* isolates could be distinguished based on isozyme typing (Sargeaunt et al., 1978). In the following years, studies of clinical epidemiology (Gathiram and Jackson, 1985), genomic DNA (Tannich et al., 1989), antigenic differences (Petri et al., 1990) and ribosomal RNA (Clark and Diamond, 1991) confirmed that *E. histolytica* and *E. dispar* should be differentiated at the species level. Based on the accumulated evidence, Diamond and Clark redescribed *E. histolytica* in 1993 and separated it from *E. dispar* (Diamond and Clark, 1993). They emphasized that *E. dispar* and non-pathogenic *E. histolytica* are synonymous. In 1997, the World Health Organization endorsed the concept of two morphologically identical *Entamoeba* species with different pathogenicity, i.e. *E. histolytica* is the pathogenic species only causing invasive disease, while *E. dispar* is a non-pathogenic species causing no disease (WHO, 1997).

### Drug development

Since the mid-19th century, ipecac, a drug produced from the dried root of the ipecacuanha plant (*Carapichea ipecacuanha*), has been widely used in India in the treatment of dysentery (Knight, 1980). In 1909, the American physicians Sidney Kohn Simon (1878–1936) and George Dock (1860–1951) independently advocated the use of salol-coated pills of ipecac in the treatment of amoebic dysentery (Dock, 1909; Simon, 1909). One year later, the British tropical medicine specialist Leonard Rogers (1868–1962) reported on the post-operation treatment of amoebic liver abscess with ipecac (Rogers, 1910). Inspired by the observation that emetine, the main constituent of ipecac, displays potent amoebicidal activity *in vitro* (Vedder, 1912), Rogers successfully treated both intestinal and hepatic amoebiasis with injectable salts of this isoquinoline alkaloid (Rogers, 1912). In 1916, the English pharmacologist and physiologist Henry Hallett Dale (1975–1968) tested the combination of emetine with bismuth, which had been previously reported by the Canadian physician William Edgar Deeks (1866–1931) to be an excellent remedy as bismuth-milk-saline for treating amoebic dysentery (Deeks, 1914), in entamoebiasis patients with promising results (Dale, 1916). The emetine-bismuth-iodide combination was widely accepted by British physicians for the treatment of amoebic dysentery for many years (Imperato, 1981).

**Figure 4.**
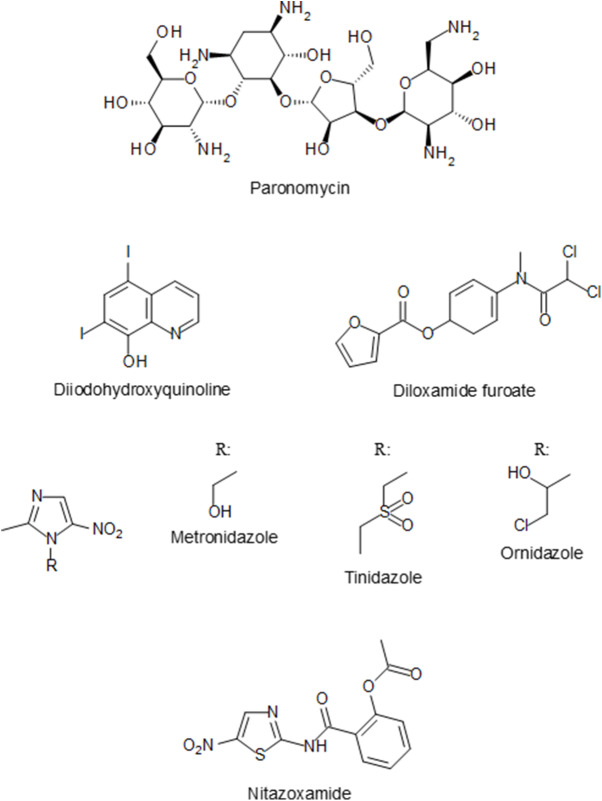
Chemical structure of currently licensed drugs for the treatment of *E. histolytica* infections.

In the following years, several other compounds were tested for their amoebicidal activity with different effectiveness, including arsenicals (acetarsol (acetarsone), carbasone, thiocarbazone and glycobiarsol (bismuth glycolylarsanilate; milibis)), halogenated hydroxyquinolines (iodohydroxyquinoline, diiodohydroxyquinoline (diodoquin) and iodochlorhydroxyquin (clioquinol)), the 4-aminoquinoline chloroquine and the 9-aminoacridine mepacrine (quinacrine; atabrine). Antibiotics were also investigated for their activity against *E. histolytica*, but most were non-specific or not very effective (Imperato, 1981). One of the antibiotics with amoebicidal activity was paromomycin (Figure 4), an aminoglycoside antimicrobial produced by the bacterium *Streptomyces rimosus*, that was found in 1959 to be effective in treating entamoebiasis (Lopez Elias and Oliver-González, 1959). Paromomycin and diiodohydroxyquinoline (Figure 4) are still used today for intraluminal carriage eradication (Chou and Austin, 2023). A third luminal anti-amoebic drug is diloxanide furoate (Figure 4), which was developed in 1956 and has been in use since the 1960s (McAuley et al., 1992). Another antibiotic that is active against *E. histolytica* is the nitro-imidazole metronidazole (Figure 4). Its effectiveness as an amoebicidal agent was demonstrated in 1966 in both intestinal and extraintestinal entamoebiasis (Powell et al., 1966). The derivatives tinidazole and ornidazole (Figure 4) were shown in the 1970s to display similar amoebicidal activity as metronidazole (Arnold, 1978). The anti-amoebic activity of the related nitro-thiazole nitazoxanide (Figure 4) was discovered in the 1990s (Gilles and Hoffman, 2002). All four nitro compounds are presently approved as first-line treatments for intestinal entamoebiasis and amoebic liver abscess (Chou and Austin, 2023).

### Current situation

Entamoebiasis remains a public health issue in many world regions, particularly in developing countries of the tropics and subtropics where poor hygiene and sanitation are a problem (Shirley et al., 2018). The highest prevalence rates of *E. histolytica* infections are in parts of Africa, Asia and Latin America. In Africa, the highest prevalence rates of intestinal entamoebiasis were reported for the East African countries of Kenya (2012: 58.3%), Rwanda (2020: 54.5%) and Uganda (2012: 19.9%) (Nasrallah et al., 2022). In Asia, high proportions of people affected by entamoebiasis were found in the Gaza Strip (2005: 69.6%), the United Arab Emirates (2009–2011: 30%), in India (2011: 11.7%) and in 7 provinces of China (2001–2004: 11.1%) (Nasrallah et al., 2022). Latin American countries with high prevalence rates of *E. histolytica* infections in certain regions include Mexico (2006–2007: 42%) and Brazil (2004, 2008 and 2018: 14.3%, 13.9%, 13.4%) (Shirley et al., 2018; Nasrallah et al., 2022). However, the reported prevalence rates of entamoebiasis are probably overestimated in many cases because of the limitation to microscopically discriminate between the pathogenic species *E. histolytica* and the non-pathogenic species *E. dispar*.

In 2019, the burden of entamoebiasis was over 2.5 million DALYs (disability-adjusted life years), and the global age-standardized DALY rate of the disease (ASDALYR) was 36.77/100 000 (Fu et al., 2023). Whereas ASDALYRs of entamoebiasis in high, middle-high and middle-income countries were quite low (0.80–10.34/100 000), the rate in low-middle and low-income countries was significantly higher (42.95–100.47), with Eastern sub-Saharan African countries having the highest ASDALYRs (114.64/100 000). The high ASDALYRs of Eastern sub-Saharan African countries are due to the high prevalence rates of entamoebiasis in these regions (see above). In terms of age groups, children under the age of 5 had the highest ASDALYRs (257.43/100 000) (Fu et al., 2023). Despite a declining global trend of ASDALYRs from 1990 to 2019, the high-income regions of North America and Australasia showed an increasing trend in the same period (Fu et al., 2023).

Although the prevalence of entamoebiasis in developed countries is generally low, the disease has increased among returning travellers and immigrants in recent years (Shirley et al., 2018). In addition, amoebic dysentery is one of the leading causes of infectious diarrhoea among holidaymakers coming back from *E. histolytica* endemic regions. The GeoSentinel Surveillance Network found that 12.5% of infectious gastrointestinal diseases diagnosed in returning travellers were due to entamoebiasis, with a rate of 14 per 1000 returned travellers (Swaminathan et al., 2009). People travelling to South America, the Middle East and South Asia have the highest risks of acquiring an infection with *E. histolytica* (Swaminathan et al., 2009).

Another group of individuals residing in developed countries that have an increased risk of contracting entamoebiasis are men who have sex with men (Shirley et al., 2018). Sequence analysis suggested that in these cases, *E. histolytica* is transmitted from person to person (Hung et al., 2008). Additionally, it has been reported that HIV-positive men who have sex with men have a higher risk of invasive amoebic disease (Hung et al., 2008; Watanabe et al., 2011).

## Conclusion

Contrary to the previous belief that *E. histolytica* is a primitive eukaryotic organism lacking classical eukaryotic organelles, this pathogen has in fact evolved more recently. Furthermore, paleoparasitological research indicates that *E. histolytica* has been a parasite of humans for a long time. This can be deduced from the detection of *E. histolytica* at a few pre-Columbian archaeological sites, which suggests that the parasite was most likely introduced into the New World by the Palaeolithic hunter-gatherers peopling the American continent about 15 000 years ago. Thus, it can be hypothesized that *E. histolytica* may have been a parasite of humans for more than 15 000 years, suggesting that the amoeba may even be an heirloom parasite. However, to determine this with certainty, further evidence of the presence of *E. histolytica* in additional samples from various ancient pre-Columbian sites is needed.

Although *E. histolytica* was established as the causative agent of amoebic dysentery and liver abscess by the end of the 19th century, it remained a puzzle for a long time why some patients developed clinical symptoms while others did not. The initial idea that there are pathogenic and non-pathogenic strains of *E. histolytica* was not an implausible suggestion. Meanwhile, it is known that harmless gut commensals can have pathogenic strains. A prominent example would be the bacterium *Escherichia coli*. By the 1980s, it was finally established that there are 2 morphologically indistinguishable amoeba species to be found in the human intestine: the pathogenic *E. histolytica* and the harmless *E. dispar*. As unusual as the existence of 2 morphologically indistinguishable *Entamoeba* species may seem, there are other examples of pathogenic species that cannot be morphologically differentiated but cause diverse disease symptoms, such as human pathogenic *Leishmania* species. Noteworthy is the fact that based on rRNA sequences, the genetic distance between *E. histolytica* and *E. dispar* is similar to that between humans and mice (Clark and Diamond, 1991).

Although entamoebiasis occurs worldwide, the majority of the disease is recorded in low-income countries in regions with poor sanitation, leading to contaminated water supply. More recently, however, imported entamoebiasis cases have increasingly been identified in non-endemic countries.

## Data Availability

The original contributions presented in the study are included in the article.
